# Non-convulsive Status Epilepticus in a Patient With Schizoaffective and Seizure Disorder on Clozapine and Electroconvulsive Therapy: A Case Report

**DOI:** 10.7759/cureus.25337

**Published:** 2022-05-25

**Authors:** Jacob R Weiss, Lauren P Baker

**Affiliations:** 1 Department of Psychiatry and Behavioral Science, Temple University Hospital, Philadelphia, USA; 2 Department of Psychiatry, Virginia Commonwealth University, Richmond, USA; 3 Department of Psychiatry and Psychology, Mayo Clinic, La Crosse, USA

**Keywords:** serious mental illness, antiepileptic drug, electroconvulsive therapy, cytochrome p450, carbamazepine, seizure disorder, schizophrenia spectrum illness, schizoaffective disorder, clozapine, non-convulsive status epilepticus

## Abstract

There is limited literature on electroconvulsive therapy (ECT) in patients with a severe schizophrenia spectrum illness and concomitant seizure disorder. In addition, it is unclear whether it is safe to perform ECT in a patient with these comorbidities and a history of status epilepticus.

This is the case of a 48-year-old patient with a history of schizoaffective disorder, bipolar type, refractory psychosis on clozapine and ECT, and seizure disorder on carbamazepine. She presented to the emergency department with suspected post-ECT delirium four days after her last ECT treatment, was found to be in non-convulsive status epilepticus, and was admitted to the neuroscience intensive care unit. Coma induction was required for seizure control. As she stabilized, her psychosis worsened, and she required psychiatric hospitalization.

Multiple factors may have contributed to the development of status epilepticus in this patient. She was on clozapine, which has a time- and dose-dependent risk of seizure that prescribers should be wary of. She had also been prescribed the antiepileptic drug carbamazepine, which induces clozapine and itself, decreasing their effectiveness. Upon the patient’s discharge, ECT was suspended indefinitely due to concern that it may have led to status epilepticus. However, case reports suggest that intractable seizures following ECT are rare.

We found no reports of status epilepticus occurring more than 60 minutes after the completion of ECT. If the benefits of ECT are significant, then it should remain a treatment option for the patient.

## Introduction

Status epilepticus is a condition characterized by a single seizure lasting more than 30 minutes or repeated seizures without an interim return of consciousness [1]. These are described as non-convulsive when there is minor or no observable motor evidence of the seizure [1,2]. Electroconvulsive therapy (ECT) is a procedure in which an electrical stimulus is delivered to induce a controlled seizure in the patient. It is currently used in the treatment of severe major depression, catatonia, acute mania, and treatment-resistant schizophrenia, often in combination with antipsychotics [3].

A few published case reports suggest that status epilepticus may occur during or immediately following ECT [2,4]. However, there is limited literature describing the occurrence of status epilepticus in the days following ECT or in the pharmacological treatment of refractory psychotic illness. Thus, it is unclear whether it is safe to perform ECT in a patient with a history of psychosis and status epilepticus.

Clozapine is a unique atypical antipsychotic that is used to treat refractory schizophrenia spectrum illnesses. While it decreases the release of dopamine in the mesolimbic pathway, it is also hypothesized to exert its antipsychotic effect by altering glutamatergic neurotransmission [5-7]. It weakly blocks dopamine subtype two (D2) receptors but saturates a lower percentage of these receptors, decreasing the risk of extrapyramidal symptoms compared to other antipsychotics [8,9]. Clozapine’s severe anticholinergic profile is responsible for many of its side effects, such as sedation, orthostatic hypotension, and constipation. Rarer and more severe effects include neutropenia, myocarditis, and seizures. These are more likely to occur with rapid dose increases and high doses of the medication. Therefore, prescribing guidelines recommend initiating a low dose of clozapine and slowly titrating the dose [10]. Clozapine is primarily catabolized to norclozapine by the hepatic cytochrome P450 (CYP) isoenzyme 1A2 and to clozapine-N-oxide by CYP3A4 [11,12]. Therefore, its plasma level and effects are susceptible to inducers and inhibitors of the CYP system.

In this article, we present the case of a patient with refractory psychosis on both clozapine and ECT, and a remote history of a seizure disorder, who was found to be in non-convulsive status epilepticus several days after receiving ECT. We discuss the relationship between ECT and seizures, the complexity of planning further treatment of refractory psychosis in a patient with seizures on both clozapine and antiepileptic drugs (AEDs), and the need for further research on the risk of status epilepticus after ECT.

## Case presentation

The patient is a 48-year-old female with a history of schizoaffective disorder and seizure disorder who presented to the emergency department with three days of altered mental status. Four days prior, she had been discharged to a group home after hospitalization for psychotic decompensation. The patient was on maintenance ECT and had received a cluster of four bitemporal treatments during the hospitalization. Her last ECT procedure was four days prior to presentation, and there had been no changes in treatment parameters. The duration of the seizure was 34 seconds by electromyogram and 46 seconds by electroencephalogram (EEG). No complications were noted, and her mentation was at baseline upon discharge. In the emergency department, the patient appeared confused, so psychiatry was consulted for "post-ECT delirium" versus decompensated psychosis. On psychiatric examination, the patient was disoriented with fluctuating attention, intermittent hand and verbal automatisms, and notably absent paranoid delusions. Neurology consultation was recommended due to concern for subclinical seizures.

The patient’s medical history included hypertension, hyperlipidemia, type II diabetes, adrenal adenoma, and a seizure disorder that had been treated with carbamazepine for 20 years. While there was a history of convulsive seizures, prior EEG monitoring was not available. There was no known history of status epilepticus. Her psychiatric history included schizoaffective disorder, bipolar type, per Diagnostic and Statistical Manual of Mental Disorders, Fifth Edition (DSM-5) criteria. Psychotic symptoms begin in childhood with persecutory delusions, auditory hallucinations, and multiple suicide attempts. Her previous psychiatric hospitalizations included stays lasting several years at state hospitals. Prior treatments included oral and long-acting injectable typical and atypical antipsychotics, various augmentation strategies, and many courses of ECT. Treatment at the time of presentation included clozapine 400 mg daily for psychosis and carbamazepine 200 mg twice daily for seizures. These medications were managed in her group home.

Routine EEG showed prolonged intervals of generalized spikes and semirhythmic activity with occasional, brief periods of attenuation lasting 10-20 seconds. The electrographic activity was indicative of non-convulsive status epilepticus, so the patient was admitted to the neurology intensive care unit for seizure management. The carbamazepine level on admission was subtherapeutic at 1.8 mg/L. Loading doses of fosphenytoin, levetiracetam, topiramate, and sodium valproate were given, but status epilepticus persisted. She was continued on maintenance AED treatment without sufficient response. She was ultimately intubated and sedated with midazolam, then placed on a pentobarbital drip with continuous EEG monitoring. After two days of induced coma, epileptic activity on the EEG ceased, and pentobarbital was slowly weaned. She was subsequently extubated and transitioned to oral topiramate and sodium valproate. Complications including drug rash, urinary tract infection, and pneumonia were addressed. As she stabilized medically, symptoms of paranoia, auditory hallucinations, and suicidal ideation returned, and she was transferred to inpatient psychiatry for further care. 

During the psychiatric hospitalization, medication regimen changes included initiating long-acting injectable haloperidol for psychotic symptoms, reducing the total dose of clozapine from 400 mg to 300 mg daily to lower the risk of seizures, increasing the dose of sodium valproate to 750 mg three times daily, and indefinitely suspending ECT due to concerns about her recent seizures.

In the following year, her psychosis worsened, resulting in multiple acute psychiatric hospitalizations. During one hospitalization, she was admitted for three months after a suicide attempt via intentional ingestion of a sharp object prompted by paranoid delusions. Upon discharge, her medication list included daily sodium valproate 1250 mg and topiramate 350 mg for seizure prophylaxis; haloperidol 20 mg, olanzapine 20 mg, and clozapine 300 mg for psychosis; and fluoxetine 20 mg for depression augmentation. ECT was not resumed.

## Discussion

Though the patient’s presentation was attributed to ECT, we question whether it contributed to the development of status epilepticus. Existing case reports suggest that, rarely, status epilepticus may occur with ECT, a form of iatrogenic seizure [2,4,13-15]. However, in these cases, status epilepticus only occurred during or within 60 minutes of completing the ECT treatment in patients with risk factors for lowered seizure threshold, including a seizure disorder, recent reduction in the dose of an AED or benzodiazepine, or concomitant use of antipsychotics and antidepressants [2]. This patient had several risk factors that made her vulnerable to developing this complication, but EEG monitoring during ECT treatment showed resolution of the seizure. She had returned to baseline mentation following ECT and only became delirious in the days after her discharge. Repeated ECT treatments like those in maintenance ECT actually increase the overall seizure threshold so that higher power or multiple stimulations are sometimes necessary to elicit a seizure during treatment [2,13]. This patient received maintenance ECT for two years, followed by a total of four treatments just prior to the occurrence of status epilepticus.

Clozapine, however, reduces the seizure threshold, increasing the annual risk of seizures in a time-dependent manner. The cumulative incidence of seizures increases from between 1.3% and 2.8% after six months to 10% after 3.8 years of treatment with clozapine [11,16,17]. Prescribers should be wary of this increasing risk the longer a patient is on clozapine. A positive correlation between dose and seizure incidence is described in the literature, but Varma et al. did not find this relationship to be statistically significant and suggested that clozapine plasma levels are better correlated [10]. Current proposed guidelines suggest monitoring clozapine drug levels and clinical signs and symptoms of seizures rather than using primary prophylaxis for clozapine-associated seizures [16]. After a second seizure occurrence on clozapine, prophylaxis with an AED is generally recommended, though particular drugs are not specified [10-11,16,18].

In this case, carbamazepine was used for seizure control for over 20 years due to a remote history of seizures. This is of particular concern since carbamazepine decreases its own circulating levels through autoinduction of CYP3A4, thereby reducing its antiepileptic effect. Since clozapine is metabolized by the same hepatic enzyme, carbamazepine also decreases clozapine’s plasma levels and its antipsychotic effect through induction of the CYP system [16,19]. Likely for this reason, the level of carbamazepine was reduced, and seizure prophylaxis was subtherapeutic at the time status epilepticus occurred. Alternate AEDs that have fewer drug-drug interactions and more stable circulating levels include sodium valproate, lamotrigine, topiramate, and gabapentin. Since sodium valproate is an effective AED in idiopathic generalized epilepsy, has a lower risk of bone marrow suppression, and has the least interference with clozapine’s metabolism, it may be a better AED option for a patient undergoing this treatment regimen [10,11,16]. These choices may enable a patient to stay on effective antipsychotic treatments.

The question of whether this patient should receive ECT in the future is complex. Factors such as the occurrence of status epilepticus, treatment of psychosis with clozapine, and a history of epilepsy requiring AEDs heighten the concern for the risk of complications and seizures. This report is limited by the little-known history of the patient’s seizure disorder. In addition, there is a paucity of reports of ECT in patients with chronic severe mental illness and concomitant seizure disorder, but those that exist conclude that ECT is likely a safe treatment option in this population [15,20]. ECT raises the seizure threshold even in patients with epilepsy, and most can be treated with ECT without lowering their dose of AEDs [2,4]. In fact, ECT has been an effective treatment option for refractory status epilepticus [4,15,21]. Per Lunde et al., patients with epilepsy on AED therapy had adequate induced seizures with ECT and effective reduction of psychiatric symptoms as well as subsequent seizure frequency [15].

Case reports suggest that seizures following ECT are rare and usually associated with other risk factors. We found no reports of status epilepticus occurring greater than 60 minutes after ECT. These factors converge on the difficult question of whether ECT should be considered in a patient who has had status epilepticus. If the benefits from ECT are significant, then it may be an appropriate option to consider when other approaches are ineffective. 

It is important to consider the severity of this patient’s chronic, treatment-resistant schizoaffective disorder and her positive response to prior ECT treatments. After consistent maintenance ECT during an extended hospitalization, her psychotic symptoms were controlled enough for her to be discharged from a state hospital to live in a group home. With ECT, she went two full years without hospitalization until the inpatient stay just prior to her presentation in the emergency department. After ECT was suspended, she was hospitalized multiple times, and several psychotropic medications, including two antipsychotics, were added to her regimen in an attempt to manage her worsening symptoms. The risk of uncontrolled psychosis for such patients is severe (self-harm, suicide attempts, and institutionalization). When ECT has significantly improved a patient’s quality of life in the past and there is supporting evidence, although limited, that ECT is safe in patients with a seizure history, ECT should remain a potential treatment option for them.

## Conclusions

Non-convulsive status epilepticus may rarely present following ECT. However, in this patient with severe schizophrenia spectrum illness on clozapine and comorbid epilepsy requiring AEDs, it can likely be attributed to alternative etiologies. Under the appropriate therapeutic AED regimen and close monitoring of clozapine levels, the patient may still benefit from ECT in the future. There is some evidence supporting the use of ECT in patients with a seizure history, but literature specifically about its use in those with a history of status epilepticus is limited to a few case reports and theoretical reasoning. More data are needed to guide safe treatment for patients with refractory psychotic illness and comorbid seizure disorder, particularly when considering ECT in patients with a history of status epilepticus.
